# Supplementary material for: An open source and reduce expenditure ROS generation strategy for chemodynamic/photodynamic synergistic therapy
Source: Nat Commun. 2020 Apr 8;11:1735. doi: 10.1038/s41467-020-15591-4 (PMC7142144; doi:10.1038/s41467-020-15591-4)
Supplement: Supplementary file 1 — Supplementary Information [file 41467_2020_15591_MOESM1_ESM.pdf]

An “Open Source and Reduce Expenditure” ROS Generation Strategy for Chemodynamic/Photodynamic Synergistic Therapy

Liu et al.

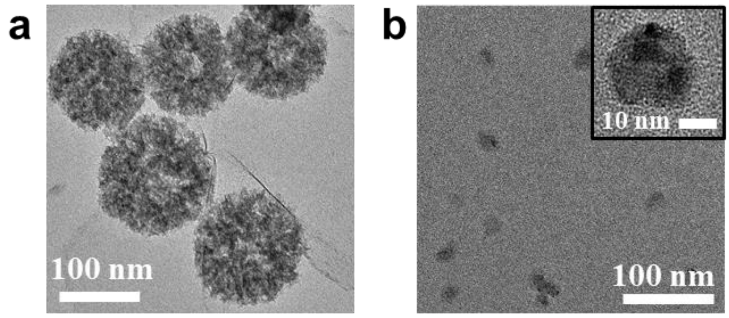

Supplementary Figure 1 TEM images of (a) MSNs and (b) CaO_2 NPs.

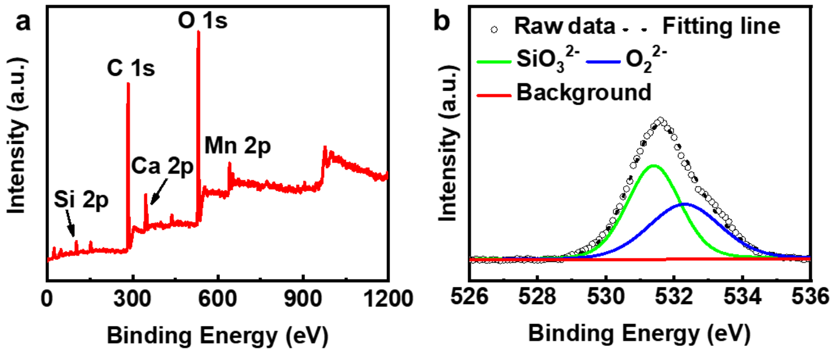

Supplementary Figure 2 (a) Survey XPS spectrum and (b) high-resolution O 1s XPS spectra of MSNs@CaO_2.

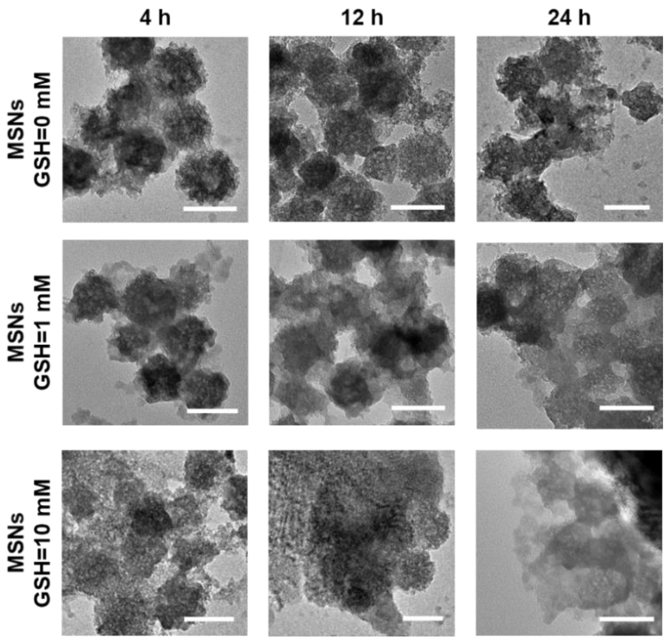

Supplementary Figure 3 The effect of GSH (0, 1, 10 mM) on biodegradation of MSNs (100 μg mL^{-1}) in PBS (10 mM, pH 7.4) for various periods of time. Scale bar: 100 nm.

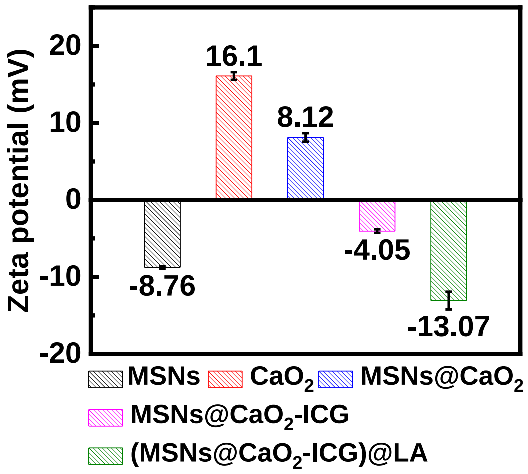

Supplementary Figure 4 Surface zeta potential of MSNs, CaO_2, MSNs@CaO_2, MSNs@CaO_2–ICG and (MSNs@CaO_2-ICG)@LA in methanol. Data are presented as mean ± SD (n = 3).

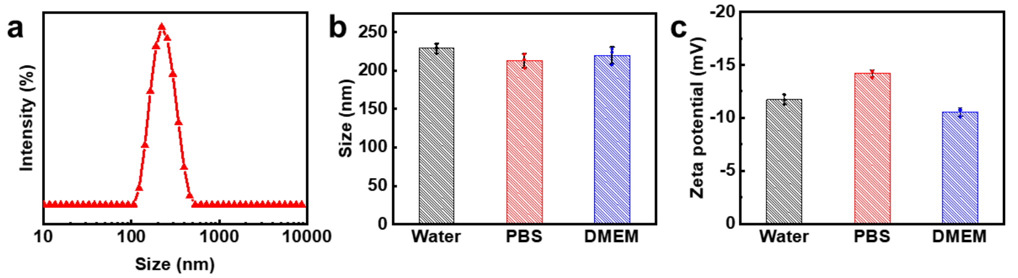

Supplementary Figure 5 (a) DLS characterization of (MSNs@CaO_2-ICG)@LA in water. (b) DLS characterization and (c) surface ζ potential of (MSNs@CaO_2-ICG)@LA in different mediums overnight. Data are presented as mean ± SD (n = 3).

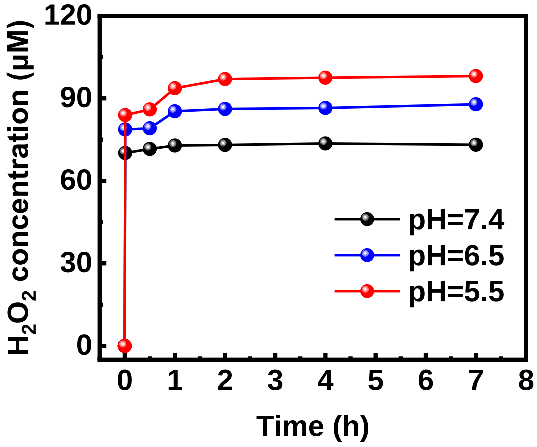

Supplementary Figure 6 H_2O_2 cumulative release from CaO_2 under different pH concditions. ([CaO_2]=10 μg mL^{-1}).

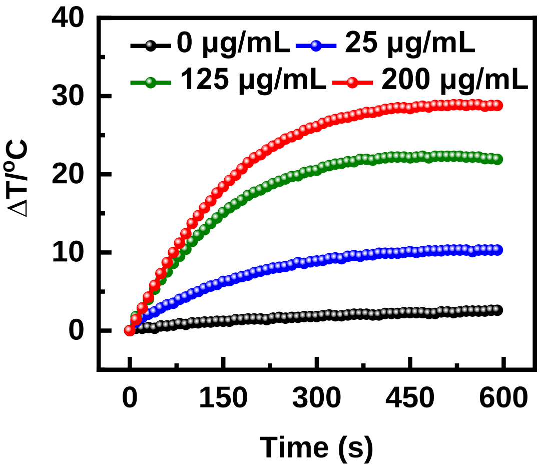

Supplementary Figure 7 Temperature measurement of (MSNs@CaO_2-ICG)@LA at concentrations of 0, 2, 10 and 20 µg mL^{-1} of ICG in water during 808 nm NIR laser irradiation (0.64 W cm^{-2}, 10 min).

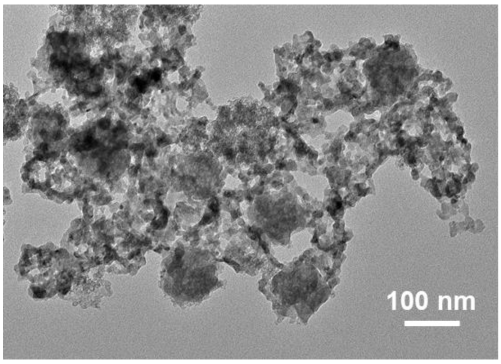

Supplementary Figure 8 The TEM image of NIR laser-irradiated (MSNs@CaO_2-ICG)@LA (Laser: 808 nm, 0.64 W cm^{-2}, 10 min).

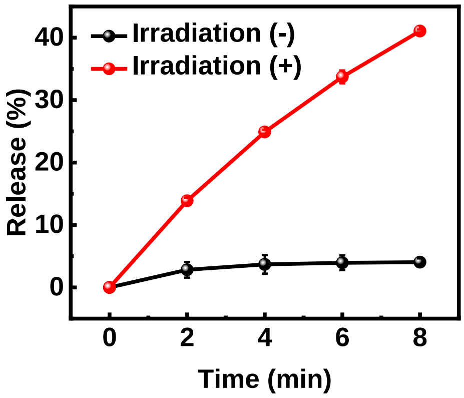

Supplementary Figure 9 ICG cumulative release profile of (MSNs@CaO_2-ICG)@LA with or without NIR irradiation (Laser: 808 nm, 0.64 W cm^{-2}, 10 min). Data are presented as mean ± SD (n = 3).

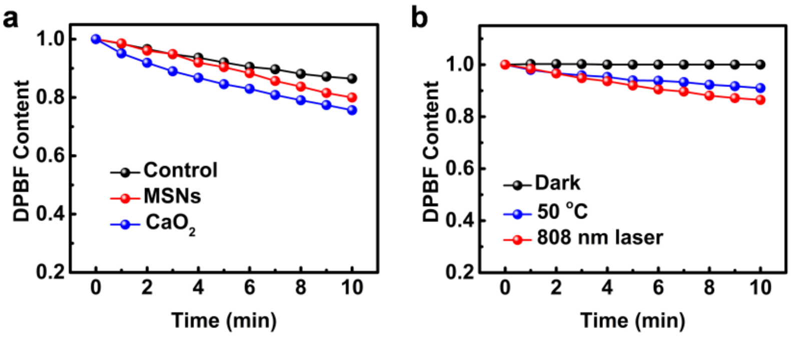

Supplementary Figure 10 (a) Time-dependent degradation of DPBF irradiated by laser for 10 min. ([MSNs] = 50 μg mL^{-1}, [CaO_2] = 50 μg mL^{-1}). (b) The effect of temperature and laser irradiation on DPBF degradation. Laser: 808 nm, 0.64 W cm^{-2}, 10 min.

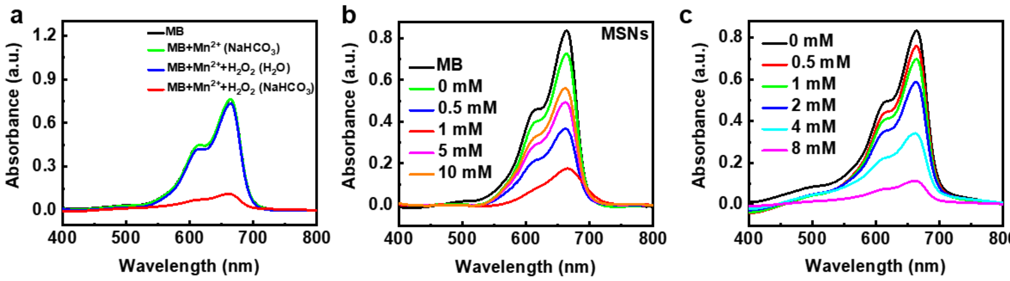

Supplementary Figure 11 (a) UV-vis absorption spectra of MB degradation in different solutions [Mn] = 0.5 mM, [H_2O_2] = 8 mM, [$NaHCO_3$/5% CO_2] = 25 mM). (b) MB degradation by ·OH generated from different concentration of GSH-treated MSNs (100 μg mL^{-1}) plus H_2O_2 (8 mM) and (c) different concentration of H_2O_2-treated MSNs (100 μg mL^{-1}) plus GSH (1 mM). [$NaHCO_3$/5% CO_2] = 25 mM.

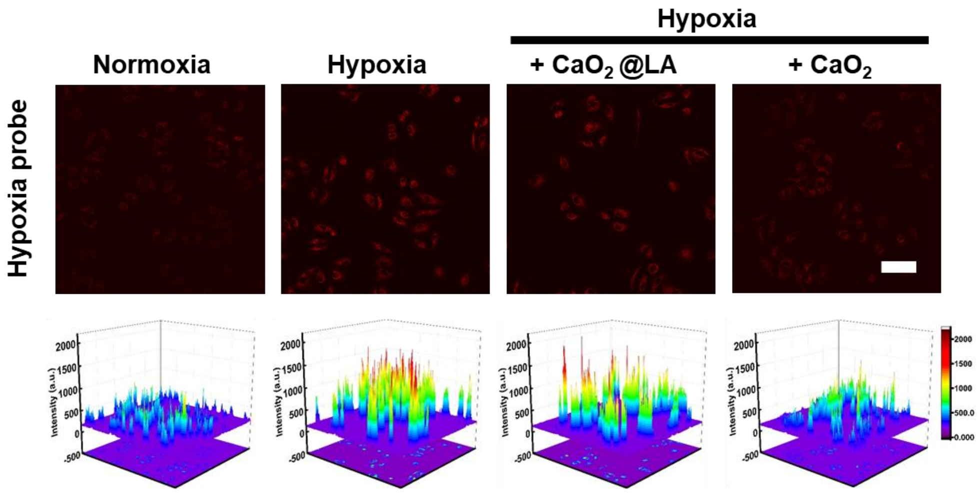

Supplementary Figure 12 CLSM images of hypoxia level in MCF-7 cells and corresponding surface plot images, respectively. Scale bar: 100 μm.

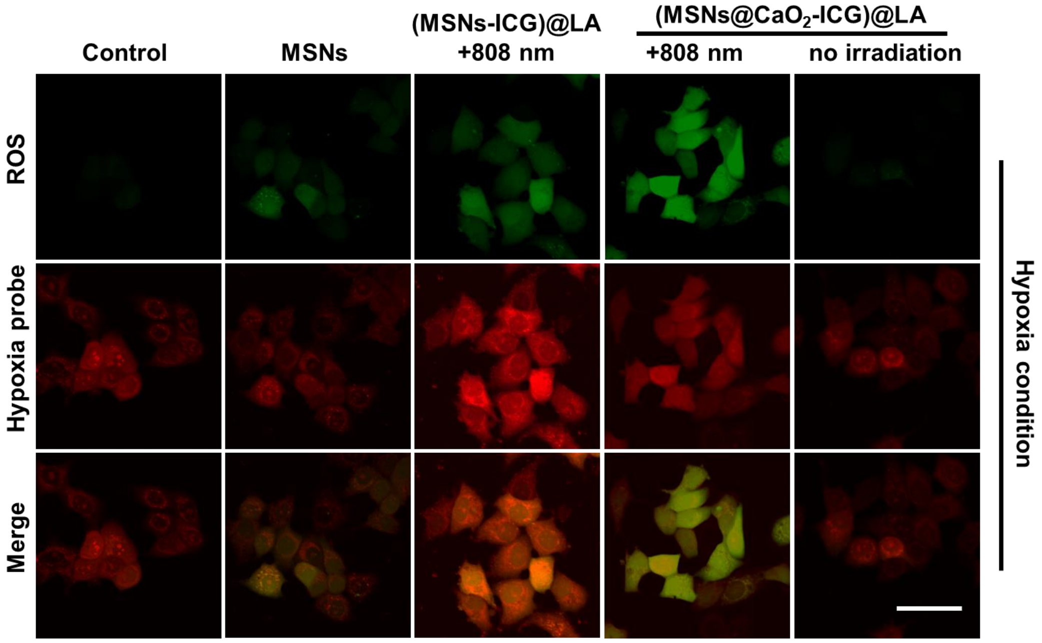

Supplementary Figure 13 Fluorescence images showing ROS and hypoxia level in MCF-7 cells with different treatment under hypoxia condition. ([MSNs]=25 μg mL^{-1}. Laser: 0.64 W cm^{-2}, 10 min). Scale bar: 50 μm.

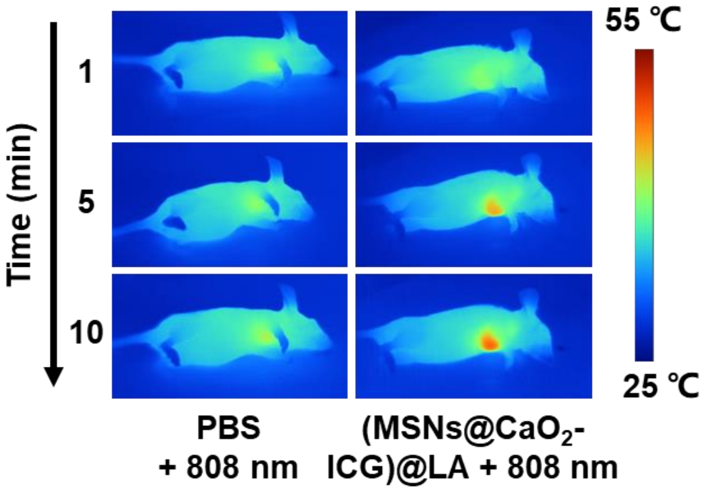

Supplementary Figure 14 IR thermal images of the MCF-7 tumor-bearing mice in groups of PBS- or (MSNs@CaO_2-ICG)@LA treated-mice under irradiation (([MSNs]= 5 mg kg^{-1}; laser: 808 nm, 0.64 W cm^{-2}, 10 min).

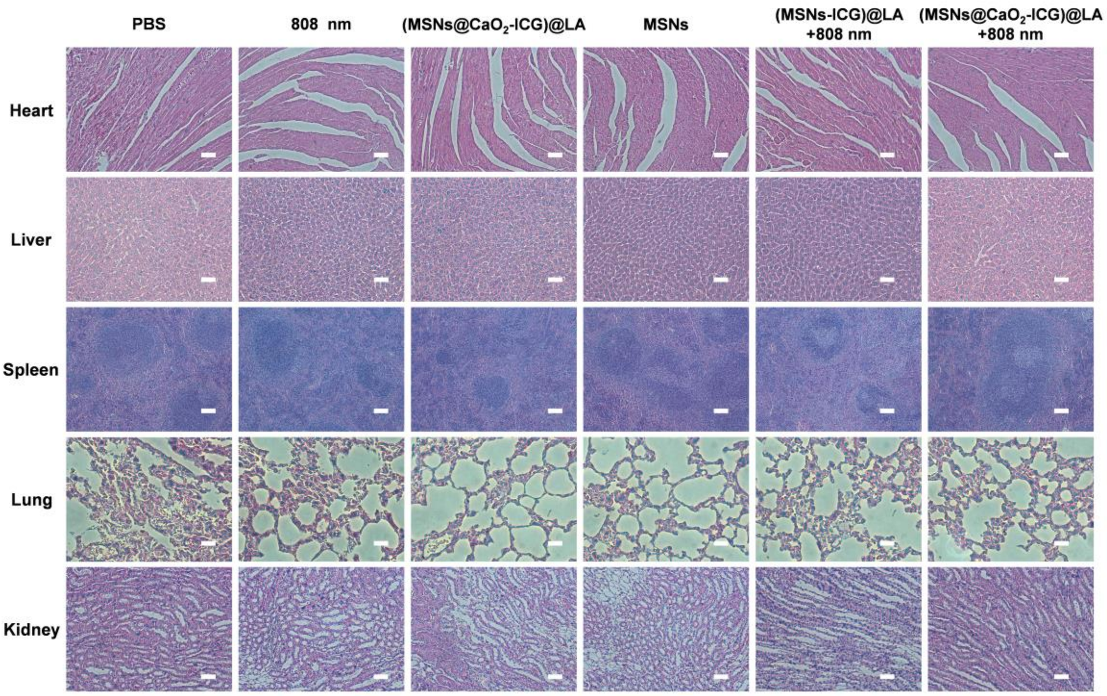

Supplementary Figure 15 H&E-stained images of organs obtained from mice in each group corresponding to Fig. 5f at 14 days. Scale bar: 100 μm.

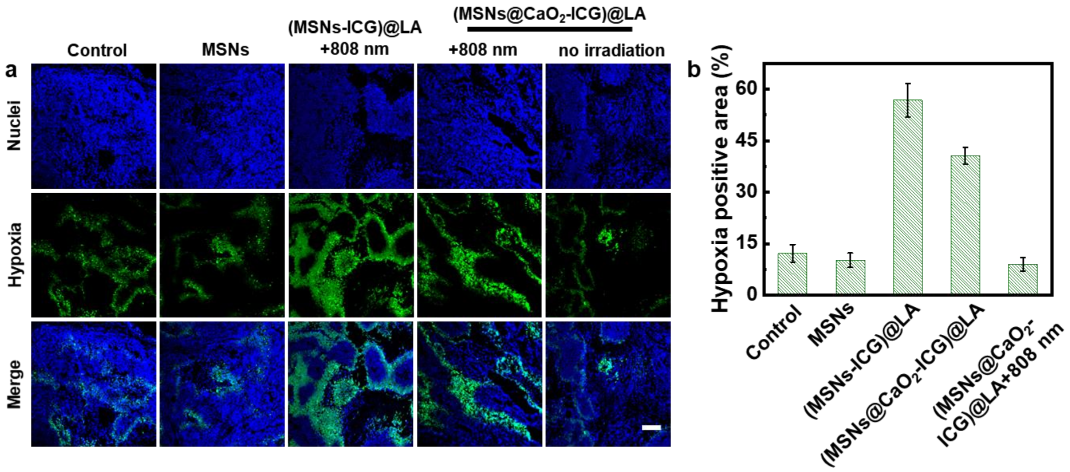

Supplementary Figure 16 (a) Immunofluorescence images of tumor slices stained by the hypoxia probe. The nuclei and hypoxia areas were stained by DAPI (blue) and anti-pimonidazole antibody (green), respectively. Scale bar: 200 μm. (b) Quantification of hypoxia area of tumor slices according to (a). Data are presented as mean ± SD (n = 3).

Supplementary Table 1. Measurement of cumulative Mn by ICP-MS in different system after reaction for 1 h.

Sample	MSNs		$(MSNs@CaO_2$-ICG)@LA	
Treatments	+ 0 mM GSH	+ 10 mM GSH	+ 10 mM GSH	+ 10 mM GSH + 808 nm laser
Released Mn (mM)	0.026	0.44	0.019	0.46
Released Mn (%)	5.20	87.98	3.74	92.27

The initial concentration of Mn in all samples was 0.5 mM.
