# Supplementary material for: An open source and reduce expenditure ROS generation strategy for chemodynamic/photodynamic synergistic therapy
Source: Nat Commun. 2020 Apr 8;11:1735. doi: 10.1038/s41467-020-15591-4 (PMC7142144; doi:10.1038/s41467-020-15591-4)
Supplement: Supplementary file 3 — Reporting Summary [file 41467_2020_15591_MOESM3_ESM.pdf]

nature research

Corresponding author(s): DBPR NCOMMS-19-35045C

Last updated by author(s): Mar 12, 2020

Reporting Summary

Nature Research wishes to improve the reproducibility of the work that we publish. This form provides structure for consistency and transparency in reporting. For further information on Nature Research policies, see Authors & Referees and the Editorial Policy Checklist .

Statistics

For all statistical analyses, confirm that the following items are present in the figure legend, table legend, main text, or Methods section.

n/a	Confirmed	
☐	☒	The exact sample size (*n*) for each experimental group/condition, given as a discrete number and unit of measurement
☐	☒	A statement on whether measurements were taken from distinct samples or whether the same sample was measured repeatedly
☐	☒	The statistical test(s) used AND whether they are one- or two-sided<br>*Only common tests should be described solely by name; describe more complex techniques in the Methods section.*
☐	☒	A description of all covariates tested
☐	☒	A description of any assumptions or corrections, such as tests of normality and adjustment for multiple comparisons
☐	☒	A full description of the statistical parameters including central tendency (e.g. means) or other basic estimates (e.g. regression coefficient) AND variation (e.g. standard deviation) or associated estimates of uncertainty (e.g. confidence intervals)
☒	☐	For null hypothesis testing, the test statistic (e.g. *F*, *t*, *r*) with confidence intervals, effect sizes, degrees of freedom and *P* value noted<br>*Give P values as exact values whenever suitable.*
☒	☐	For Bayesian analysis, information on the choice of priors and Markov chain Monte Carlo settings
☒	☐	For hierarchical and complex designs, identification of the appropriate level for tests and full reporting of outcomes
☒	☐	Estimates of effect sizes (e.g. Cohen's *d*, Pearson's *r*), indicating how they were calculated

Our web collection on statistics for biologists contains articles on many of the points above.

Software and code

Policy information about availability of computer code

Data collection	NovoExpress software was used to acquire flowcytometry data. The cell fluorescence imaging was collected by FV1200, Olympus.
Data analysis	Confocal fluorescence microscopy imaging data was analyzed by FV1200, Olympus (Japan). Flowcytometry data was analyzed by NovoExpress (ACEA NovoCyte). And also the ImageJ, Origin, Microsoft Excel 2016.

For manuscripts utilizing custom algorithms or software that are central to the research but not yet described in published literature, software must be made available to editors/reviewers. We strongly encourage code deposition in a community repository (e.g. GitHub). See the Nature Research guidelines for submitting code & software for further information.

Data

Policy information about availability of data

All manuscripts must include a data availability statement. This statement should provide the following information, where applicable:

- Accession codes, unique identifiers, or web links for publicly available datasets
- A list of figures that have associated raw data
- A description of any restrictions on data availability

The authors declare that the data supporting the findings of this study are available within the paper [and its supplementary information files], or from the corresponding author on reasonable request.

Field-specific reporting

Please select the one below that is the best fit for your research. If you are not sure, read the appropriate sections before making your selection.

Life sciences study design

All studies must disclose on these points even when the disclosure is negative.

Sample size	The sample size of synthesized (MSNs@CaO2-ICG)@LA nanoparticle was determined by the transmission electron microscopy (TEM) images. We randomly measured the size of five nanoparticles and used the average as the sample size.
Data exclusions	No data were excluded from the analyses.
Replication	All experiments were repeated and experimental findings were reproducible.
Randomization	The cells and animal used in this paper were randomly distributed into several groups for the further experiments when the cells were in logarithmic growth or tumor volumes reached the proper size .
Blinding	The technician collected and analyzed data without knowing the group assignment for each sample.

Reporting for specific materials, systems and methods

We require information from authors about some types of materials, experimental systems and methods used in many studies. Here, indicate whether each material, system or method listed is relevant to your study. If you are not sure if a list item applies to your research, read the appropriate section before selecting a response.

Materials & experimental systems

n/a	Involved in the study
☐	☒ Antibodies
☐	☒ Eukaryotic cell lines
☒	☐ Palaeontology
☐	☒ Animals and other organisms
☒	☐ Human research participants
☒	☐ Clinical data

Methods

n/a	Involved in the study
☒	☐ ChIP-seq
☐	☒ Flow cytometry
☒	☐ MRI-based neuroimaging

Antibodies

Antibodies used	Anti-pimonidazole monoclonal antibody, Hypoxyprobe-1 Plus Kit, Hypoxyprobe Inc.
Validation	Validation of each antibody was done under standard information offered by the supplier.

Eukaryotic cell lines

Policy information about cell lines

Cell line source(s)	The human MCF-7 breast cancer cells, human A549 adenocarcinoma alveolar basal epithelial cells and NHDF normal human dermal fibroblasts cells were purchased from Cell Bank, the Committee of Type Culture Collection of Chinese Academy of Sciences.
Authentication	Cells were routinely tested for mycoplasma contamination using MycoSET Mycoplasma real-time PCR detection Kit (Life Technologies, Foster City, CA, USA).
Mycoplasma contamination	These cell lines have passed the conventional tests of cell line quality control methods including morphology, isoenzymes, and mycoplasma.
Commonly misidentified lines (See ICLAC register)	None of these cell lines were used.

Animals and other organisms

Policy information about studies involving animals; ARRIVE guidelines recommended for reporting animal research

Laboratory animals	Female Balb/c mice (4 weeks) were purchased from Beijing Vital River Laboratory Animal Technology Co., Ltd.
Wild animals	No wild animals.
Field-collected samples	No field-collected samples.

Ethics oversight	All the animal experiments were conducted in accordance with the guidelines of the Department of Laboratory Animal Science of Peking University Health Science Center. All animal experiments were conducted and agreed with the Institutional Animal Care and Use Committee of the Beijing Institute of Basic Medical Science (Beijing, China).

Note that full information on the approval of the study protocol must also be provided in the manuscript.

Flow Cytometry

Plots

Confirm that:

- [x] The axis labels state the marker and fluorochrome used (e.g. CD4-FITC).
- [x] The axis scales are clearly visible. Include numbers along axes only for bottom left plot of group (a 'group' is an analysis of identical markers).
- [ ] All plots are contour plots with outliers or pseudocolor plots.
- [x] A numerical value for number of cells or percentage (with statistics) is provided.

Methodology

Sample preparation	MCF-7 cells were seeded on culture dishes at a density of 1 × 105, respectively. After incubation for 24 h, the cells were treated with samples and incubated for 2, 4 or 6 h at 37 °C. Then, the cells were washed with cold PBS, harvested and analyzed immediately using flow cytometry (ACEA NovoCyte, Agilent Bio.). The mean fluorescence intensity of 3 × 104 cells was recorded for each sample.
Instrument	ACEA NovoCyte, Agilent Biosciences
Software	The supporting software NovoExpress was used to collect and analyze the flow cytometry data.
Cell population abundance	The absolute cells around 10000 were analyzed for each group.
Gating strategy	*Describe the gating strategy used for all relevant experiments, specifying the preliminary FSC/SSC gates of the starting cell population, indicating where boundaries between "positive" and "negative" staining cell populations are defined.*

- [ ] Tick this box to confirm that a figure exemplifying the gating strategy is provided in the Supplementary Information.
